# How Do People Perform an Inspection Time Task? An Examination of Visual Illusions, Task Experience, and Blinking

**DOI:** 10.5334/joc.123

**Published:** 2020-09-30

**Authors:** Yke Bauke Eisma, Joost de Winter

**Affiliations:** 1 Delft University of Technology, Delft, NL

**Keywords:** Attention, Response accuracy, Response speed

## Abstract

In the inspection time (IT) paradigm, participants view two lines of unequal length (called the Pi-figure) for a short exposure time, and then judge which of the two lines was longer. Early research has interpreted IT as a simple index of mental speed, which does not involve motor activity. However, more recent studies have associated IT with higher-level cognitive mechanisms, including focused attention, task experience, and the strategic use of visual illusions. The extent to which these factors affect IT is still a source of debate. We used an eye-tracker to capture participants’ (*N* = 147) visual attention while performing IT trials. Results showed that blinking was time-dependent, with participants blinking less when the Pi-figure was visible as compared to before and after. Blinking during the presentation of the Pi-figure correlated negatively with response accuracy. Also, participants who reported seeing a brightness illusion had a higher response accuracy than those who did not. The first experiment was repeated with new participants (*N* = 159), enhanced task instructions, and the inclusion of practice trials. Results showed substantially improved response accuracy compared to the first experiment, and no significant difference in response accuracy between those who did and did not report illusions. IT response accuracy correlated modestly (*r* = 0.18) with performance on a short Raven’s advanced progressive matrices task. In conclusion, performance at the IT task is affected by task familiarity and involves motor activity in the form of blinking. Visual illusions may be an epiphenomenon of understanding the IT task.

## Introduction

Inspection Time (IT) is defined as “the time required by a subject to make a single observation or inspection of the sensory input on which a discrimination of relative magnitude is based” (Vickers & Smith, 1986), or less formally, “the minimum time required to tell the difference between two perceptually different things” (Irwin, 1984, p. 47). In the standard IT paradigm, the participant views two vertical lines of different lengths, connected by a horizontal line at the top. The participants are exposed to this so-called Pi-figure for a brief time and subsequently have to indicate which of the two lines, the left or the right one, was the longer one. IT is then defined as the exposure time for which participants achieved a threshold accuracy level (e.g., 90% correct). Alternatively, performance at an IT task can be defined as the percentage of trials that were answered correctly (e.g., Ritchie, Bates, Der, Starr, & Deary, 2013).

In a meta-analysis of 92 studies, Grudnik and Kranzler (2001) estimated the mean IT-IQ correlation at –0.30, or –0.51 after correcting for attenuation and range restriction. Early research has theorized that IT scores are an index of mental speed, and therefore a valid indication of psychometric intelligence (Brand, 1981; Brand & Deary, 1982). Jensen (2006) argued that IT is a sensitive index of the “speed of perceptual intake” (p. 84) because participants merely have to determine the difference in a visual stimulus, with no need for providing an immediate motor response as would be the case in, for example, reaction time tasks. Elsewhere, Kranzler and Jensen (1989) mentioned: “IT, the only index of mental speed that does not involve either motor (output) components or executive cognitive processes (metaprocesses), is held to tap individual differences in the ‘speed of apprehension,’ the quickness of the brain to react to external stimuli prior to any conscious thought.” (pp. 329–330). Similarly, Gregory, Nettelbeck, Howard, and Wilson (2008) argued that IT could be used as a biomarker for cognitive decline because an IT task, unlike a reaction time task, is free from psychomotor confounding and does not involve a speed-accuracy trade-off. According to Deary (2000), IT is the simplest possible index that shows a strong correlation (|*r*| > 0.3) with IQ.

Stankov (2004) lamented that “even today some writings on IT, particularly by the ardent supporters of biological interpretations of intelligence, sound like the author(s) believe it is synonymous with intelligence” (p. 351). The current consensus, however, is that to equate a performance measure (IT) with mental speed would be an oversimplification, and that the mechanisms of association between IT and intelligence differences are far from fully understood (Deary, 2001). Structural equation models of Johnson and Deary (2011), for example, suggest that IT may have no unique relationship to general intelligence, and that IT is just one of the elementary cognitive tasks in the broader structure of cognitive ability.

One possible reason for IT not being a pure index of mental speed and intelligence is that IT may be affected by higher-level cognitive mechanisms. According to Deary and Stough (1996), the possibility of IT being a consequence of intelligence differences would represent a validity threat of the IT paradigm: “the inspection-time measure would lose much of its apparent attraction for intelligence researchers, because it would become just another task that clever people perform well” (p. 603).

Several types of cognitive mechanisms for performing IT tasks have been reported in the literature. First, about 50% or more of participants report using cues from visual illusions to perform better at the IT task (e.g., Alexander & Mackenzie, 1992; Chaiken & Young, 1993; Egan & Deary, 1992; Egan, 1994; and see Grudnik & Kranzler, 2001 for a meta-analysis). The two most commonly reported illusions in the IT task are the apparent movement illusion, where people perceive the shorter of the two lines of the Pi-figure to grow as it is overlaid by the mask, and the flash brightness illusion, where people see a bright flash originating from the shorter of the two lines (Alexander & Mackenzie, 1992; Simpson & Deary, 1997). A number of studies have shown that participants who report using illusions perform substantially better at the IT task than nonusers (Egan & Deary, 1992; Egan, 1994; Mackenzie & Bingham, 1985; Mackenzie & Cumming, 1986). Various authors have examined whether different types of masks prevent the perception of illusions and accordingly increase the validity of the IT measurement (e.g., Evans & Nettelbeck, 1993; Stough, Bates, Mangan, & Colrain, 2001), or whether the mask is needed at all (for further discussion, see Egan, 1993).

The second type of cognitive mechanism concerns the effects of experience and practice. It is well established that performance on neuropsychological tests, such as tests of memory and attention, improve with experience (e.g., Seibel, 1963; Sullivan et al., 2017). For IT tasks as well, it has been found that participants perform better if they are re-tested (Anderson, Reid, & Nelson, 2001; Blotenberg & Schmidt-Atzert, 2019; Bors, Stokes, Forrin, & Hodder, 1999; Larson, Saccuzzo, & Brown, 1994; Nettelbeck & Vita, 1992). These findings call into question the notion that IT represents an unmalleable mental quality, and suggest that IT is under the influence of executive functioning or associative mechanisms. For example, participants may come to understand how to perform the task through self-monitoring of past and current performance (Nettelbeck, 2001). The fact that the IT task is susceptible to task familiarity effects has been implicitly acknowledged by the inclusion of familiarization trials (e.g., Bors et al., 1999; Deary et al., 2004; Duan, Dan, & Shi, 2013). However, it is unknown how IT performance improves with practice.

The third type of cognitive mechanism concerns attention (e.g., Bors et al., 1999; Nettelbeck, 2001). It has been found that persons with higher IQ exhibit shorter fixations in visual search tasks than normal-IQ persons, suggesting a link between attention and IQ (e.g., Sargezeh, Ayatollahi, & Daliri, 2019). Levy (1992) presented the attention hypothesis, which states that IT reflects how well a participant sustains attention to the task. White (1996) pointed out that the micro-deployment of attention is a possible validity threat of the hypothesis that IT is a fundamental task of visual discrimination. For example, IT performance may be better for participants who are visually attentive during the task-critical moments, that is, when the Pi-figure stimulus is visible. The attention hypothesis relates to research which indicates that lapses in attention are related to working memory, executive control, and intelligence (Adam & deBettencourt, 2019; Larson & Alderton, 1990; Oberauer, 2019; Unsworth, Redick, Lakey, & Young, 2010).

So far, attention levels during IT tasks have been measured in indirect ways. Egan and Deary (1992) let participants perform an IT task concurrently with a mental arithmetic task. The participants who reported illusions for the single IT task did not report them in the dual-task condition. Noteworthily, participants who reported illusions in the single-task condition had an IT in the dual-task condition that was shorter than that of participants who did not perceive illusions in the single-task condition, suggesting that illusions are merely a by-product of good performance. Anderson (1989) let participants perform the IT task in a self- or forced-paced manner, under the assumption that self-pacing reduces distraction. In addition, he applied a fixed versus random period between the end of one IT trial and the beginning of the next and argued that attentional processes would be inhibited if the period were random. Results confirmed expectations that the random period in the forced-paced condition yielded the longest ITs. Hutton, Wilding, and Hudson (1997) let children perform a test battery that measured attentional abilities and subsequently controlled for attention by including IT together with the attention scores in a regression analysis for predicting IQ. Results showed that IT was a statistically significant predictor of IQ even when the attention scores were included in the regression model. The above studies indicate that IT is associated with attention, but do not elucidate the mechanisms of focused attention while performing an IT task.

Several studies have used physiological measures to examine how participants attend to the IT task. Nettelbeck, Robson, Walwyn, Downing, and Jones (1986) presented five experiments in which the eye-movements of low- and normal-IQ participants were measured while performing an IT task. The results showed that the low-IQ participants were prone to distraction before target onset. For example, in one of their experiments, the average number of off-target eye movements was 16.1 out of 240 trials for low-IQ participants, whereas the normal-IQ participants exhibited none. In the same experiment, the number of blinks averaged at 10% and 5% of trials for low- and normal-IQ participants, respectively, an effect that was not statistically significant. Further research on the role of attention was performed by Deary et al. (2004), who let participants perform an IT task in combination with fMRI. They found elevated activity in select regions of the brain, which they interpreted as effort-related processes and cognitive processes related to attention, working memory, imagery, and vision. Caryl (1994) found significant correlations between IT and ERPs 100 to 200 ms after the stimulus onset and noted that “perhaps ability to focus attention is the fundamental difference between individuals in this task rather than a difference in speed of perceptual intake” (p. 43). More recently, Hill et al. (2011) let a high- and low-IQ group perform an IT task while measuring their ERPs. Based on the larger N1 response for the high-IQ group, they suggested that the link between IT and IQ can be attributed to individual differences in spatial attention. The studies of Deary et al. and Hill et al. indicate that IT is a complex task in which attentional processes play a role. However, so far, it is still unknown *how* people attend to the IT task.

In summary, the validity of IT as an index of ‘low-level’ mental speed has been questioned from the perspective of three cognitive mechanisms: (1) self-reported visual illusions, (2) experience effects, and (3) attention. The extent to which these factors affect IT is presently a source of debate. This study attempts to extend the findings of previous research by examining how illusions relate to IT performance, how participants improve their IT performance as a function of trial number, and how attention relates to IT performance. Attention in this study was operationalized as ‘not blinking’, consistent with Johns, Crowley, Chapman, Tucker, and Hocking (2009), who found that reaction times were impaired when blinks occurred during the stimulus onset.

## Methods

### Participants

One hundred forty-eight MSc engineering students participated. The data for one participant were not recorded correctly. The remaining 147 participants were 45 females and 102 males with a mean age of 23.33 years (*SD* = 2.13). Twenty-two participants used contact lenses and 13 used glasses. The number of participants who wore glasses during the experiment was smaller than 13, as an undocumented number of participants were encouraged to remove their glasses to enhance the quality of the eye-tracking data.

### Apparatus

Movements of the right eye were recorded at 2000 Hz using the SR Research EyeLink 1000 Plus. Participants were asked to keep their head in the head support during the entire experiment.

The visual stimuli were presented using a computer running ‘SR Research Experiment Builder’ (version 1.10.1386) on a 64-bit Windows 7 Professional operating system. The computer contained an Intel Core i7-4790K Processor (@ 4.00 GHz) and NVIDIA GeForce GTX 970 graphics card. The stimuli were shown on a 24.5-inch BENQ monitor (XL2540) with a resolution of 1920 × 1080 pixels, and a display area of 531 × 298 mm. The refresh rate of the monitor was set to 144 Hz. The monitor was positioned 95 cm from the table edge. For a distance between the eyes and monitor of 91 cm, the monitor subtended horizontal and vertical viewing angles of 33° and 19°, respectively. The eye-tracking camera/IR light source was located at 65 cm from the head support. Participants wore closed-back headphones to block out ambient noise. There was no natural light in the room. The illuminance of the fluorescent lighting in the room near the experimental setup was around 400 lx, as measured with a Konica Minolta T-10MA illuminance meter.

### Procedures

Before the experiment, participants completed a standard EyeLink nine-dot calibration procedure. Participants first looked at a number of stimuli as part of an unrelated pupillometry study lasting about 15 min (De Winter, Petermeijer, Kooijman, & Dodou, 2020). Next, the IT experiment started. Participants received task instructions on the monitor (see Figure S1 in the supplementary materials). These instructions stated that participants needed to accurately discriminate between one short and one long bar, and mentioned that the long bar would be randomly varied between the left and right positions. Furthermore, it was mentioned that participants had to press the key that matched the position of the long bar. The correct answers were the ‘A’ key (covered with a red sticker) if the longest leg was on the left side and the ‘L’ key (covered with a blue sticker) if the longest leg appeared on the right. The instructions were accompanied with an image depicting the fixation marker and an image depicting the Pi-figure with its long leg on the left, and the text “In the above example the left bar is longer, so the correct response is ‘left’ (key with the red sticker)”. In a second instruction screen, participants were informed as follows: “This is not a reaction time task – you have as much time as you like in which to respond. You can make your response whenever you like”. The experimenter provided further explanation in case the participant had questions.

Next, participants received 80 IT stimuli. The stimuli were presented in the form of videos with a frame rate of 144 frames per second.

Each video consisted of the following parts in this order (see Figure S2 for screenshots):

– A fixation marker in the middle of the screen for 500 ms
– A blank screen for 604 ms
– The Pi-figure stimulus for 14, 21, 35, 42, 56, 83, 104, or 153 ms
– A mask for 500 ms

The stimuli were drawn in MATLAB and saved as a video file having a frame rate of 144 fps. It was verified using a 1000 Hz high-speed camera that it took 2 to 3 ms for the Pi-figure and mask to appear on the screen. Accordingly, the above exposure times of the Pi-figure were regarded as accurate. The video of the high-speed camera is available in the supplementary materials.

The legs of the Pi-figure were 124 pixels apart horizontally, which corresponds to a viewing angle of 2.2°. The short leg was 138 pixels long (2.4° vertically), and the long leg was 276 pixels long (4.8° vertically). The lines of the Pi-figure were black and 2 pixels thick. The Pi-figure was placed on a light grey background (RGB 237, 237, 237).

Although participants had been informed that they could take as much time as they wanted to respond, the maximum response time was 3.9 s (this corresponds to 5 s since the beginning of the trial minus 1.1 s, which was the elapsed time that the Pi-figure was presented). It was reasoned that this time limit would be more than sufficient for respondents to provide input.

If a participant provided a correct response, the word “CORRECT” was shown for 0.7 s, and if the participant provided an incorrect response, “INCORRECT” was shown for 0.7 s (Figure S2a). No feedback was provided if the participant did not respond.

For each of the eight exposure times, five videos showed the longer leg on the right side, and five showed the longer leg on the left side. The 80 videos were shown in a random order that was different for each participant. The experimental procedure lasted approximately 5 minutes.

After the 80 IT trials, participants answered two multiple-choice questions:

- “During the experiment I experienced (n)one of the following visual illusions:”, with the following response options:
  “1 = The shorter bar of the stimulus appeared to ‘grow’ larger after the mask appeared.”
  “2 = The mask that covered the stimulus appeared ‘brighter’ on the side where the bar was shortest.”, and
  “3 = I have experienced no illusions at all.”
- “Have you used the perceived illusion as a cue to perform the task? Choose no if no illusion was perceived)”, with the response options “1 = Yes”, and “2 = No”.

### Data Processing

Blinks were defined based on the vertical eye-gaze coordinate. Periods during which vertical eye-gaze coordinate data were unavailable, as well as periods where participants glanced above or below the edges of the screen, were labelled as ‘blinks’. A manual inspection of the raw data (pupil diameter, vertical gaze coordinates) showed that the vast majority of data losses were indeed due to blinks, rather than due to looking away from the screen. A margin of 100 ms was added before and after each blink, to account for the closing time and reopening time, respectively (Caffier, Erdmann, & Ullsperger, 2003). For each trial, data were recorded until 0.5 s after the participant provide a response. Because of the aforementioned 100 ms margin that surrounded each blink, blink data were included up to 0.4 s after the participant responded.

The following measures were calculated for each participant:

*– Non-responses*. The percentage of trials out of 80 where the participant did not respond within the allocated time.
*– Response accuracy*. The percentage of responses that were correct. Non-responses were excluded from this calculation.
*– Mean response time*. The response time was defined as the time between the onset of the Pi-figure and the moment the participant pressed one of the two keys. Non-responses were excluded from this calculation. It is noted that an IT task is not a response time task; we did not instruct participants to respond as quickly as possible. However, this does not preclude us from reporting how much time participants took to respond. We used the response time as an indicator of information processing efficiency and experience effects. Vickers, Nettelbeck, and Willson (1972) found that the mean response time decreased with exposure duration of the Pi-figure.

Note that some literature defines IT based on estimating the minimum exposure time necessary to achieve a threshold percentage of correct discriminations of the longer line (e.g., Vickers & Smith, 1986). We opted for the number of correct responses as a simpler and more tractable performance score (e.g., Posthuma, De Geus, & Boomsma, 2001; Ritchie et al., 2013). Furthermore, it was impossible to calculate a threshold percentage because some participants showed poor performance (e.g., for 27 of 147 participants, less than 60% of responses were correct).

A preliminary analysis of the horizontal and vertical eye gaze coordinates revealed no noteworthy patterns between trials in which participants provided a correct response and trials in which the participants provided an incorrect response. In short, it was found that participants, on average, looked about 15 pixels more downward at the moment of stimulus presentation for trials with an incorrect response as compared to trials with a correct response. We suspect that this small effect is confounded with partial eye closures, causing an apparent downward movement of the vertical gaze coordinate. Because eye-movement effects appeared to be small and not of general interest, they were not pursued further.

We calculated associations between the performance measures and trial number, self-reported illusion, and percentage of trials in which the participant was blinking (for distinct elapsed times during the trial: 0, 0.22, 0.44, 0.66, 0.88, and 1.10 s). Group comparisons for the illusions were performed using unequal-variances *t*-tests (Welch’s tests). Cohen’s *d* was used as an effect size measure. Associations between the response accuracy and the percentage of trials in which the participant blinked were computed at the level of participants, using two complementary measures: Pearson’s product-moment correlation coefficient (*r*) and Spearman’s rank-order correlation coefficient (ρ). Pearson’s correlation is a measure of the degree of linear association. It is intuitively interpretable but has the disadvantage of being less stable when outliers are present or when the distribution is heavy-tailed. Spearman’s correlation, on the other hand, is robust to outliers and tailed distributions (De Winter, Gosling, & Potter, 2016).

### Follow-up Experiment

The above experiment had a number of characteristics that may have made the task difficult or confusing for participants. A follow-up experiment was conducted, with the goal to examine whether the results replicated in improved experimental conditions. The follow-up experiment was the same as the above experiment, but with the following modifications:

- **New participants.** Participants were 165 MSc engineering students. Six participants had to be removed due to missing or low-quality eye-tracking data. The remaining 159 participants were 50 females and 109 males with a mean age of 23.52 years (*SD* = 1.98). Thirty-three participants used visual aids during the experiment (23 contact lenses, 10 glasses). The research was approved by the university ethics committee, and all participants provided written informed consent.
- **Enhanced instructions.** The instructions were expanded. More specifically, it was mentioned: “In each trial, you will be shown (one after the other): 1) a fixation marker, 2) then, a stimulus consisting of two lines of different lengths, 3) shortly after, the lines will be masked”. The fixation marker, stimulus (Pi-figure), and mask were each accompanied with a figure. On a second instruction screen, a Pi-figure with its long leg on the left, and a Pi-figure with its long leg on the right were shown. The accompanying text stated: “Your task is to indicate which of the two lines of the stimulus was the longest. If the left line was longer, press the left shift key. If the right line was longer, press the right shift key”.
- **Practice trials.** The first experiment did not contain practice trials. In the follow-up experiment, we included three practice trials, with exposure times of 1000 ms, 500 ms, and 201 ms. After each practice trial, the correct answer was shown on the screen.
- **Uniform luminance.** In the first experiment, the feedback screens stating “CORRECT” or “INCORRECT” were brighter than the stimulus trials (see Figure S2a). This increased brightness may have given rise to exogenous eye blinks. In the follow-up experiment, we used a uniform grey background (RGB 127, 127, 127) for the entire IT task, including the feedback screens. Furthermore, the “CORRECT” and “INCORRECT” messages were shown in an outline font to minimize the overall effect of luminance (Figure S2b).
- **Adjustments to the stimulus.** The blank screen before the presentation of the Pi-figure was shown for 500 ms instead of 604 ms to reduce participants’ waiting time. Furthermore, the lines of the Pi-figure were made thicker (6 pixels) to increase the likelihood that participants could see the Pi-figure. The legs of the Pi-figure were 90 pixels apart horizontally, which corresponds to a viewing angle of 1.6°. The short leg was 90 pixels long (1.6° vertically), and the long leg was 180 pixels long (3.1° vertically).
- **No response time limit.** In the first experiment, participants had to respond within a time limit of 3.9 s. In the follow-up experiment, participants had infinite time to respond. Because of this, non-responses were impossible, and we expected that this would reduce confusion among participants. Because long response times (i.e., outliers) were possible, the median response time, instead of the mean response time, was used as a measure.
- **Enhanced questions about illusions.** In the first experiment, we inquired whether participants perceived an apparent movement illusion or a brightness illusion. In the follow-up experiment, we asked about visual illusions in greater detail, based on Alexander and Mackenzie (1992). More specifically, we asked whether participants experienced (1) the ends of the lines of the stimulus moving/stretching downward upon the appearance of the mask, (2) a flash arising from the stimulus upon the appearance of the mask, (3) a small black gap at the ends of the stimulus lines upon the appearance of the mask, (4) another type of visual illusion, or (5) no illusion. Additionally, participants were required to answer “what exactly did you perceive, and did you use the illusions to perform better at the task?” using a textbox field.
- **Inclusion of cognitive test.** The first experiment did not include an IQ test. To examine the criterion validity of the IT task, we added a 12-item version of Raven’s advanced progressive matrices (Arthur, Tubre, Paul, & Sanchez-Ku, 1999). Participants had 7 minutes to solve as many of the 12 items as they could. The Raven’s matrices were completed after the IT task, and participants entered their responses using the keyboard.
- **Binocular eye-tracking.** We used a newer version of the EyeLink 1000 Plus eye-tracker, which measured eye-movements of both eyes at 2000 Hz. A blink was defined as in the first experiment, except that the gaze data for the two eyes were first averaged. If vertical gaze data for one of the two eyes was unavailable, then this was counted as a blink.

## Results

The 147 participants each performed 80 IT trials. On average, participants had 4.74 non-responses (*SD* = 9.88). Accordingly, the average number of responses per participant was 75.26.

### Experience Curves

**Figure 1:**
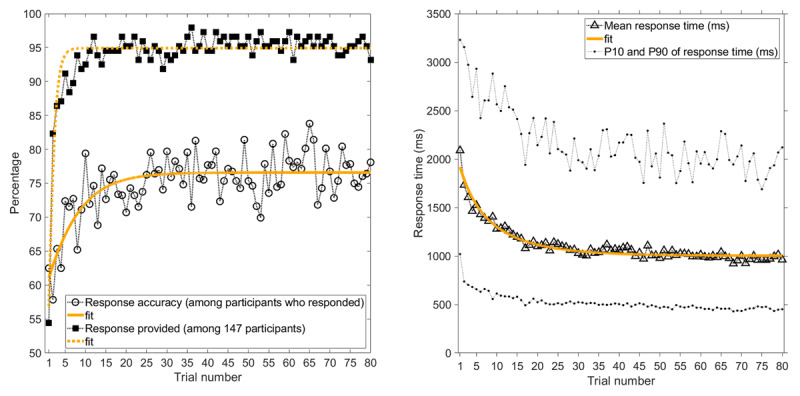
Experience curves as a function of trial number, where Trial 1 is the first IT stimulus presented, and Trial 80 is the last IT stimulus presented. Left = Response accuracy (i.e., percentage of responding participants who provided a correct response), and percentage of 147 participants who provided a response within the allocated time. Right = Mean response time of the participants who provided a response, together with 10th and 90th percentiles. Exponential fits, *y* = 1/(*a* + b*exp(–c*x)), are shown where *x* is the trial number, and *a, b*, and *c* are fitted parameters. For the ‘response accuracy’ curve, *a* = 0.0131, *b* = 0.00387, *c* = 0.155 (*r*^2^ = 0.53). For the ‘response provided’ curve, *a* = 0.0105, *b* = 0.0198, *c* = 1.033 (*r*^2^ = 0.87). For the ‘mean response time’ curve, *a* = 0.00100, *b* = –0.000516, *c* = 0.0811 (*r*^2^ = 0.94). Note that the IT stimuli were presented in random order.

Figure 1 shows the percentage of 80 trials where a response was provided within the allocated time, the response accuracy (i.e., the percentage of responses that were correct), and the mean response time. With increasing experience, response accuracy increased, response time decreased, and the likelihood of giving a response within the available time increased. The latter measure reached an early plateau at about ten trials. The mean response time, however, kept reducing with trial number (Figure 1). There were no significant associations between participants’ response accuracy and age (*r* = –0.07, *p* = 0.399) or gender (*r_pb_* = 0.14, *p* = 0.094, coded as 1 = male, 2 = female).

### Effect of Exposure Time

**Figure 2:**
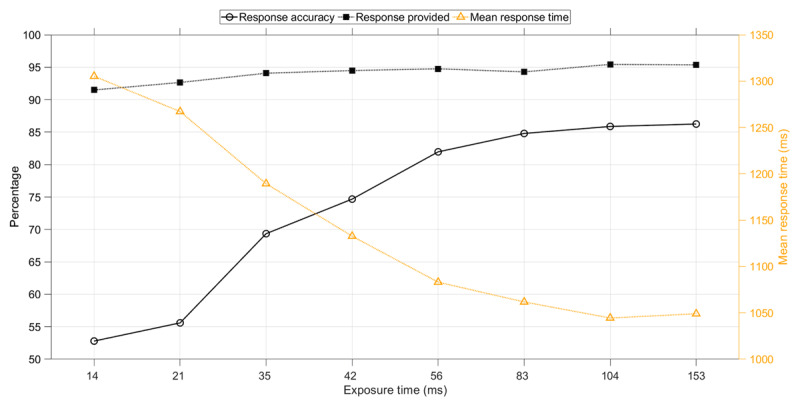
Response accuracy (i.e., percentage of responses that were correct), percentage of trials with a response within the allocated time, and mean response time as a function of exposure time of the Pi-figure. The means and standard deviations are provided in Table S1.

Longer exposure times yielded a higher response accuracy and a faster response (Figure 2). More specifically, when the exposure time was low (14 ms), response accuracy was barely above chance level, and when the exposure time was high (153 ms), the response accuracy was 86.2%. The mean response time decreased from 1305 ms for a 14 ms exposure time to 1049 ms for a 153 ms exposure time. Participants also were more likely to respond within the available time limit when the exposure time was higher.

### Association with Self-Reported Illusions

Next, we examined the association between IT performance and self-reported illusions. The brightness illusion was relatively infrequent (17 participants, 12%) as compared to the growing illusion (56 participants, 38%) and no illusion (74 participants, 50%). Of the 17 participants who experienced the brightness illusion, 14 (82%) reported using this illusion as a cue to perform the task. Of the 56 participants who experienced the growing illusion, 47 (84%) reported using this illusion as a cue to perform the task.

Results in Table 1 show that the brightness illusion is associated with a higher response accuracy, a lower percentage of non-responses, and a faster mean response time as compared to no illusion. The effects are illustrated using boxplots in the supplementary materials (Figures S3a, S4a, S5).

**Table 1:** Inspection time task performance per self-reported illusion (*N* = 147).

	Response accuracy (% of trials in which the participant responded)	No response (% of all 80 trials)	Mean response time (ms)

	Mean (*SD*)	Mean (*SD*)	Mean (*SD*)

Growing illusion (*n* = 56)	74.52 (14.23)	3.98 (5.74)	1141 (522)
Brightness illusion (*n* = 17)	80.33 (7.76)	0.82 (1.01)	860 (273)
No illusion (*n* = 74)	72.33 (16.87)	6.22 (12.81)	1202 (560)
	**Welch’s test**	**Welch’s test**	**Welch’s test**

Growing vs. no illusion	*t*(126.4) = 0.80, *p* = 0.426	*t*(106.8) = 1.33, *p* = 0.185	*t*(122.4) = 0.64, *p* = 0.523
Brightness vs. no illusion	*t*(55.3) = 2.94, *p* = 0.005	*t*(76.8) = 3.57, *p* < 0.001	*t*(51.3) = 3.68, *p* < 0.001

### Attention during the Trials

In total, 11760 trials were completed (147 participants × 80 trials per participant). A keypress response, either correct or incorrect, was recorded in 11063 of those trials. Blinking data for 19 of those 11063 trials were excluded because there were not enough ocular data. More specifically, participants in those 19 trials were observed to be blinking for over 50% of the time of that trial, which could be explained because of poor eye tracking quality or participants not looking at the screen.

**Figure 3:**
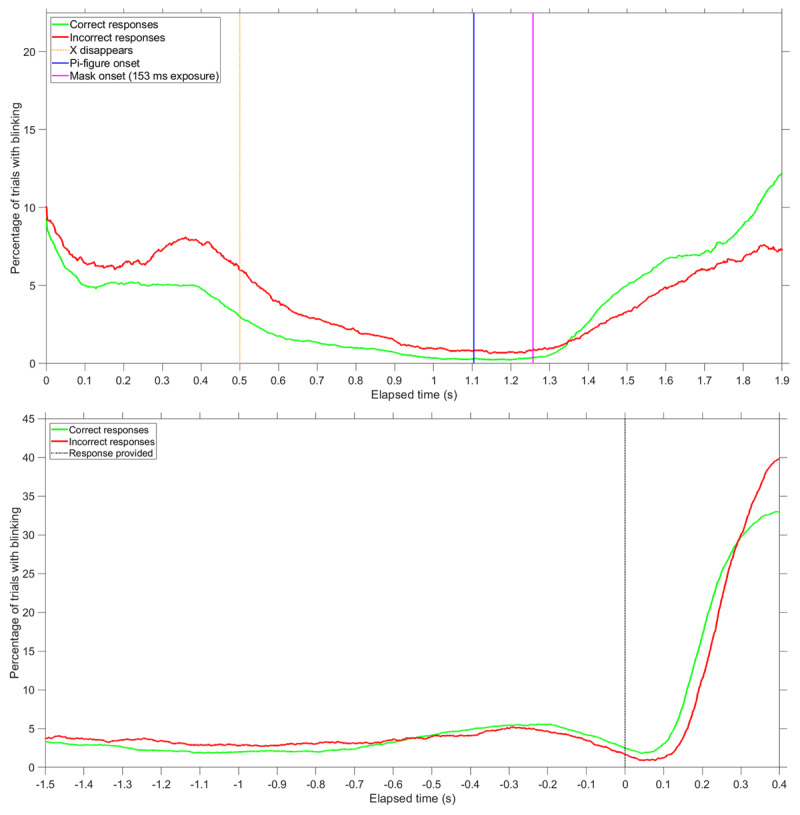
Percentage of trials in which participants were blinking, for each time sample. A distinction is made between trials where participants provided a correct response (*n* = 8308) and trials where participants provided an incorrect response (*n* = 2736). Top figure: results time-locked to the stimulus (occurring at *t* = 1.1 s). Vertical lines are shown for the moment the fixation marker (X) disappeared, the moment the Pi-figure was presented, and the moment the mask was presented for the maximum exposure time of 153 ms. Bottom figure: results time-locked to the participants’ response, indicated by the vertical line at t = 0 s. Participants were provided with a “CORRECT” or “INCORRECT” feedback message after responding. Data were included up to 0.4 s after the participant provided a response; therefore, the number of data points near the end of the top figure (*t* = 1.9 s) or the beginning of the bottom figure (*t* = –1.5 s) is reduced (*n* = 8163 for correct responses, *n* = 2705 for incorrect responses).

Figure 3 shows the percentage of trials with blinking as a function of elapsed time during the IT trial. A distinction is made between 8308 trials in which a participant provided a correct response and 2736 trials in which the participant provided an incorrect response.

Two main patterns can be distinguished. First, the blinking patterns were highly dynamic. Participants hardly blinked during the crucial period of the presentation of the Pi-figure, and they blinked after the trial had ended (Figure 3, top). Second, there is a distinction between blinking patterns of correct and incorrect responses. Incorrect responses were associated with blinking when the Pi-figure was presented, whereas correct responses were associated with blinking afterwards (Figure 3, top).

The high blink rates for correct responses after the presentation of the Pi-figure can be explained by the fact that participants responded about half a second faster for correct responses compared to incorrect responses (*M* = 982 ms for the 8322 correct responses, *M* = 1461 ms for the 2741 incorrect responses). As can be seen in Figure 3, bottom, many participants blinked after having responded.

**Figure 4:**
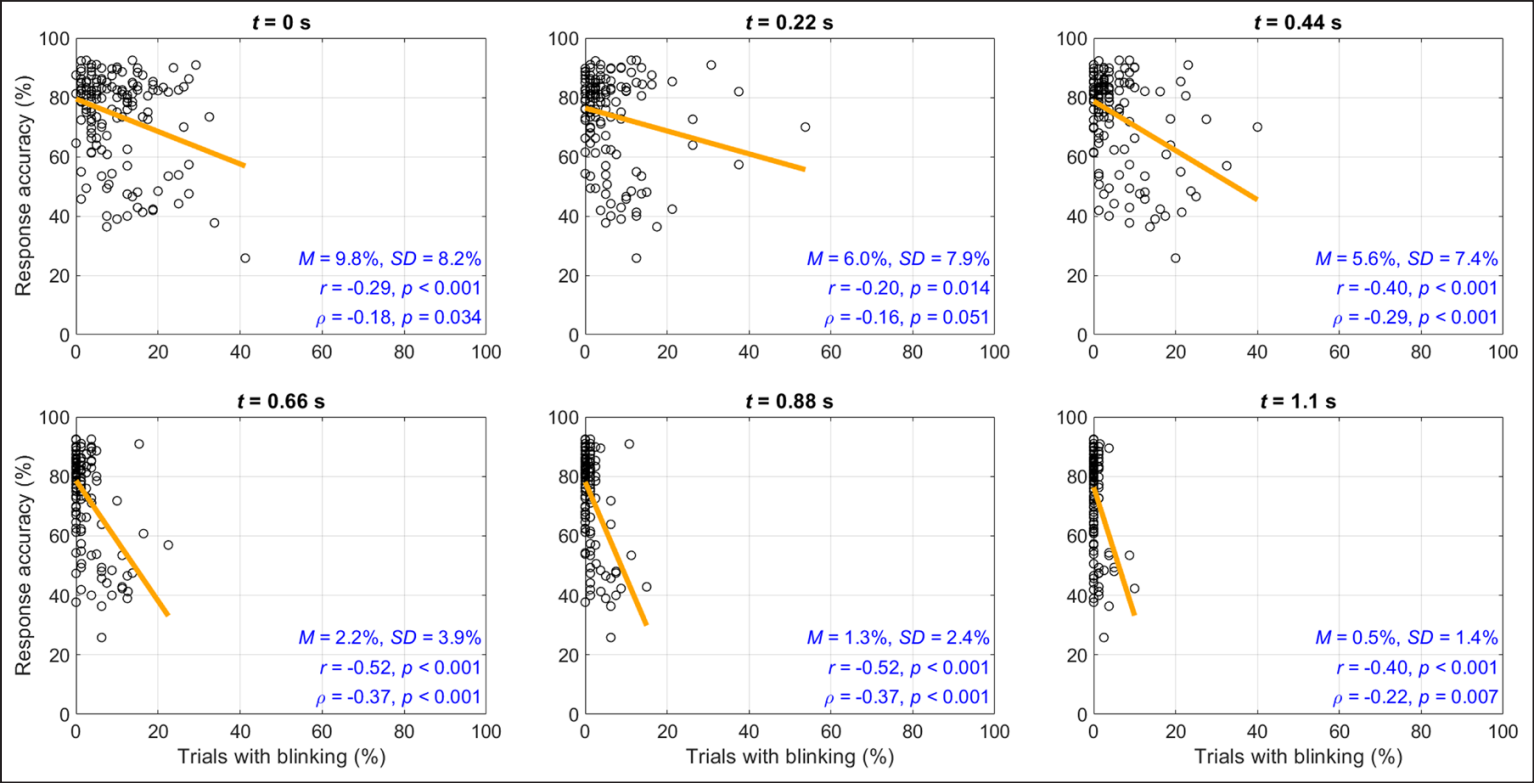
Response accuracy (i.e., percentage of responses that were correct) versus the percentage of trials with blinking at the level of participants (*N* = 147), per 220 ms of elapsed time into the trial. Also shown is a least-squares regression line, means and standard deviations of the percentage of trials with blinking, and the Pearson correlation coefficients (*r*) and Spearman rank-order correlation coefficients (ρ). The fixation marker onset occurs at *t* = 0 s. The onset of the Pi-figure occurs at *t* = 1.1 s.

The individual differences in blinking are illustrated using scatter plots at the level of participants, see Figure 4. It can be seen that a negative correlation exists between blinking and the percentage of correct responses.

### Blinking as a Function of Trial Number

**Figure 5:**
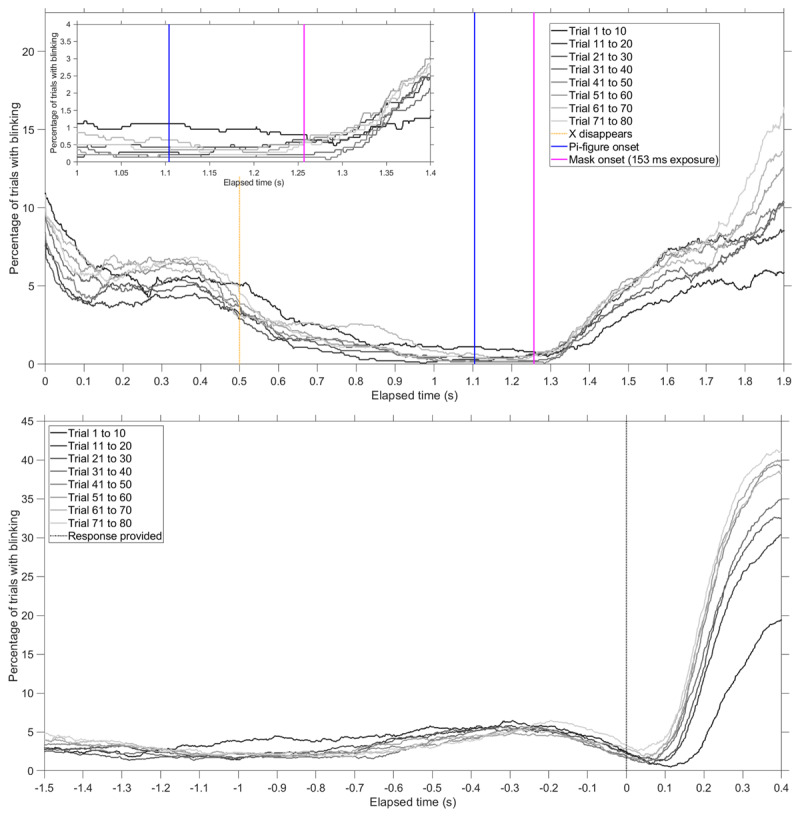
Mean percentage of all trials in which the participant was blinking, per group of 10 trials (11044 trials in total). Top: results time-locked to the stimulus (occurring at *t* = 1.1 s). An inset is provided for elapsed times between 1.0 s and 1.4 s. Vertical lines are shown for the moment the fixation marker (X) disappeared, the moment the Pi-figure was presented, and the moment the mask was presented for the maximum exposure time of 153 ms. Bottom: results time-locked to the participants’ response, indicated by the vertical line at *t* = 0 s. Participants were provided with a “CORRECT” or “INCORRECT” feedback message after responding. Data were included up to 0.4 s after the participant provided a response.

Figure 5 shows the percentage of blinking as a function of elapsed time during the IT trial. A distinction is made between the degree of task experience, by creating 8 groups of 10 trials. It can be seen that during the first ten trials, participants relatively often blinked during the presentation of the Pi-figure. At later trials, participants blinked more and more after the stimulus presentation.

### Follow-up Experiment

**Figure 6:**
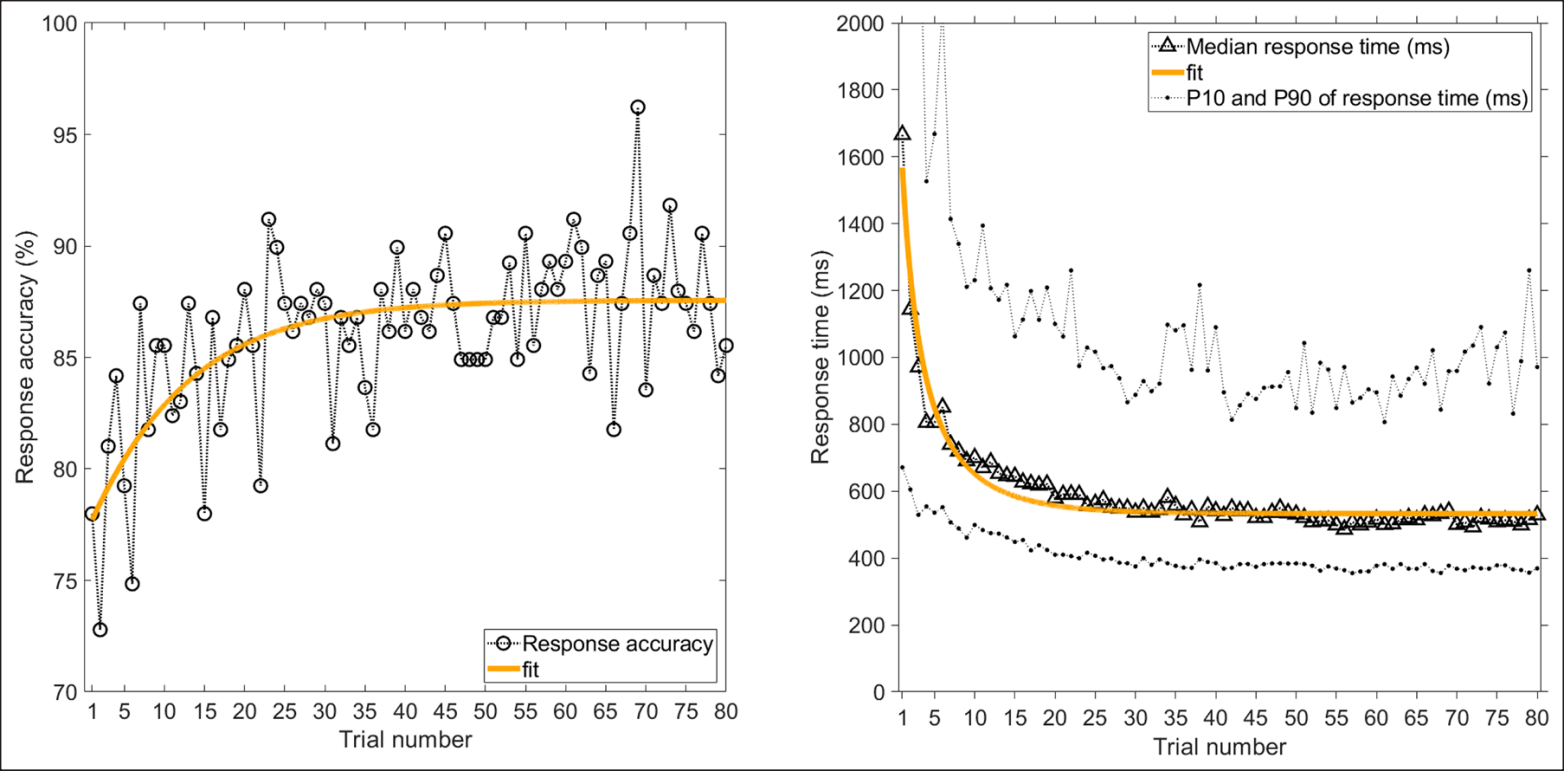
Follow-up experiment: Experience curves as a function of trial number, where Trial 1 is the first IT stimulus presented, and Trial 80 is the last IT stimulus presented. Left = Response accuracy (i.e., percentage of 159 participants who provided a correct response). Right = Median response time among 159 participants, together with 10th and 90th percentiles.

**Figure 7:**
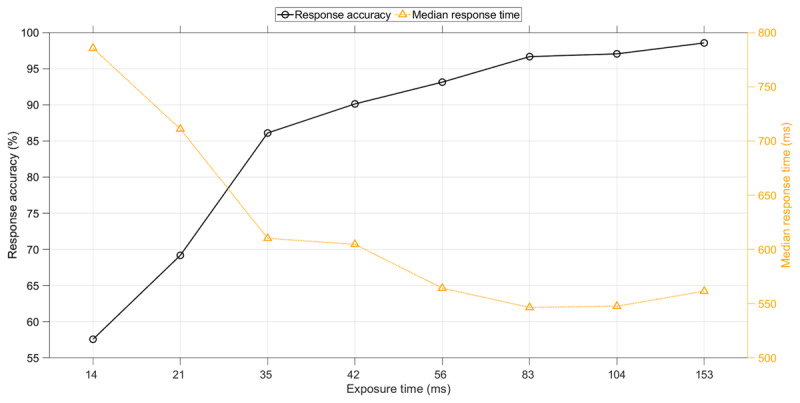
Follow-up experiment: Response accuracy and median response time as a function of exposure time of the Pi-figure. The median response time was calculated per participant per exposure time and subsequently averaged over the 159 participants. The means and standard deviations are provided in Table S2.

In the follow-up experiment, learning curves can be seen, similar to the learning curves of the first experiment, see Figure 6. For the response time, the fit was still strong (*r*^2^ = 0.95). The response accuracy, however, showed a less strong learning curve as compared to the first experiment. The response accuracy was considerably higher as compared to the first experiment, with a score of 98.6% for the highest exposure duration, compared to 86.2% in the first experiment (Figure 7). Participants also responded substantially faster, with a median response time for the highest exposure duration of 562 ms versus 958 ms in the first experiment (see Tables S1 and S2). There were no significant associations between participants’ response accuracy and age (*r* = 0.00, *p* = 0.960), and females had a slightly lower response accuracy than men (*r_pb_* = –0.18, *p* = 0.026, coded as 1 = male, 2 = female).

Exponential fits, *y* = 1/(*a* + b*exp(–c*x)), are shown, where *x* is the trial number, and *a, b*, and *c* are fitted parameters. For the ‘response accuracy’ curve, *a* = 0.0114, *b* = 0.00158, *c* = 0.0895 (*r*^2^ = 0.40). For the ‘median response time’ curve, *a* = 0.00188, *b* = –0.00143, *c* = 0.1433 (*r*^2^ = 0.95). Note that the IT stimuli were presented in random order.

In the follow-up experiment, the response accuracy and the response times were similar between participants who reported having perceived an illusion and participants who reported having perceived no illusion (see Table 2). In other words, the association between illusions and task performance, as observed in the first experiment (see Table 1), did not replicate. In fact, the results showed that participants who perceived the moving/stretching illusion had a significantly *longer* response time than those who perceived no illusion.

**Table 2:** Follow-up experiment: Inspection time task performance per self-reported illusion (*N* = 159).

	Response accuracy (% of trials)	Median responsetime (ms)

	Mean (*SD*)	Mean (*SD*)

Moving/stretching illusion (*n* = 49)	85.65 (7.76)	629 (176)
Flash illusion (*n* = 39)	84.70 (8.50)	609 (217)
Black gap illusion (*n* = 8)	87.81 (4.47)	549 (81)
Other illusion (*n* = 25)	87.04 (9.40)	530 (104)
No illusion (*n* = 38)	86.90 (6.15)	552 (129)
	**Welch’s test**	**Welch’s test**

Moving/stretching vs. no illusion	*t*(85.0) = 0.83, *p* = 0.406	*t*(84.7) = 2.33, *p* = 0.022
Flash vs. no illusion	*t*(69.3) = 1.30, *p* = 0.196	*t*(62.2) = 1.40, *p* = 0.167
Black gap vs. no illusion	*t*(13.3) = 0.49, *p* = 0.632	*t*(15.5) = 0.09, *p* = 0.932
Other vs. no illusion	*t*(37.5) = 0.07, *p* = 0.946	*t*(58.3) = 0.75, *p* = 0.457

An examination of the responses to the free-response item showed that 157 of 159 participants provided a meaningful response. The responses varied considerably, with many participants reporting no illusion, or describing general phenomena (“only experienced an effect similar to tunnel vision”) rather than illusions related to task performance. However, several interesting observations were made:

- 27 participants reported that a change occurred on the shorter side of the Pi-figure in particular, e.g. “I looked at where the moving appeared and then I knew that the other side would have the longer end”.
- 9 participants reported that the shorter line moved more slowly than the longer line, e.g. “The lines seemed to stretch downwards. The line which stretched down faster was the longer line, so yes I used it to perform the task”.
- 7 participants reported aftereffects, e.g. “I could see the lines on the screen even after the image was gone and it helped me predict some answers correctly”. However, in some cases the aftereffects were described as having a negative effect on performance, e.g. “I did, however, experience an after-image which made it difficult to identify this contrast, the more tired my eyes became”.
- 4 participants reported that a flash occurred on one side, e.g. “I saw a flash on the shorter side when the mask appears”. For three participants, the flash occurred on the shorter side; for one participant it occurred on the longer side.

It is of note that several participants reported relying on the illusion (“perceived moving stimulus, kind of amazed that I sometimes did not really see the whole stimulus but knew what side it was, left or right”) while others stated that they saw no illusion whatsoever (“I did not see any illusions, I am just really good at this”). Interesting as well, some participants reporting seeing no illusion in the multiple-choice item, but still referred to a change or motion, e.g., “stretching of line on the shorter side; therefore, the other side should have been the longest line” or “the longest line didn’t move as much … so, it was the side with little movement”.

**Figure 8:**
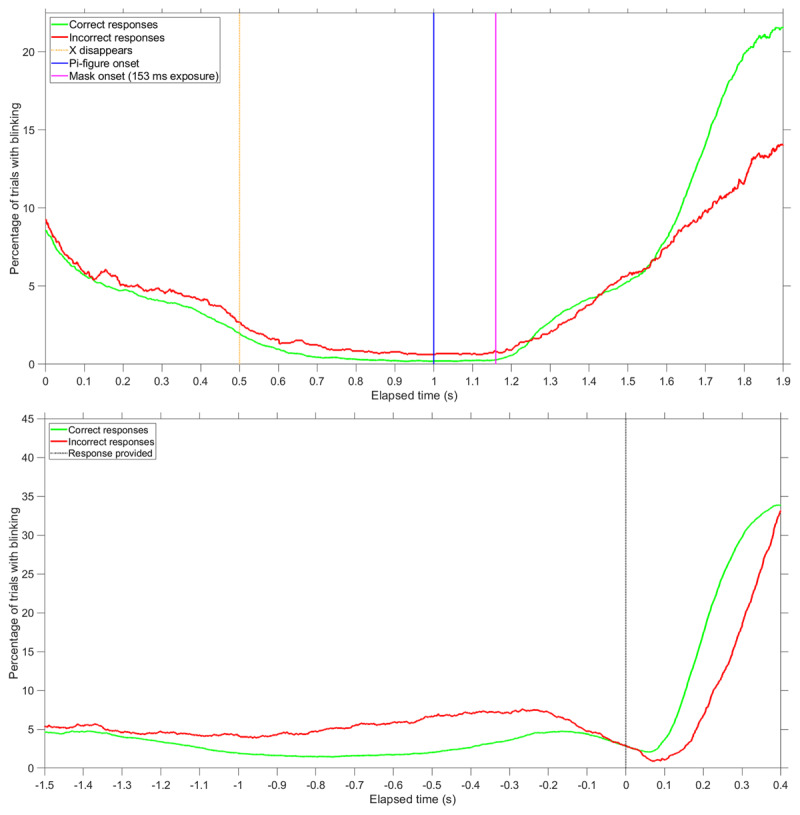
Follow-up experiment: Percentage of trials in which participants were blinking, for each time sample. A distinction is made between trials where participants provided a correct response (*n* = 10915) and trials where participants provided an incorrect response (*n* = 1768). Top figure: results time-locked to the stimulus (occurring at *t* = 1.0 s). Vertical lines are shown for the moment the fixation marker (X) disappeared, the moment the Pi-figure was presented, and the moment the mask was presented for the maximum exposure time of 153 ms. Bottom figure: results time-locked to the participants’ response, indicated by the vertical line at t = 0 s. Participants were provided with a “CORRECT” or “INCORRECT” feedback message after responding. Data were included up to 0.4 s after the participant provided a response; therefore, the number of data points near the end of the top figure (*t* = 1.9 s) or the beginning of the bottom figure (*t* = –1.5 s) is reduced (*n* = 7967 for correct responses, *n* = 1569 for incorrect responses).

In total, 12720 trials were completed (159 participants × 80 trials per participant), with response data and eye-tracking being available for 12683 trials. Eye-blinking patterns showed a similar pattern as in the first experiment, with participants avoiding blinking at the moment of the presentation of the Pi-figure, and blinking after that (Figure 8). Again, correct responses were associated with not blinking during the moment of Pi-figure presentation.

**Figure 9:**
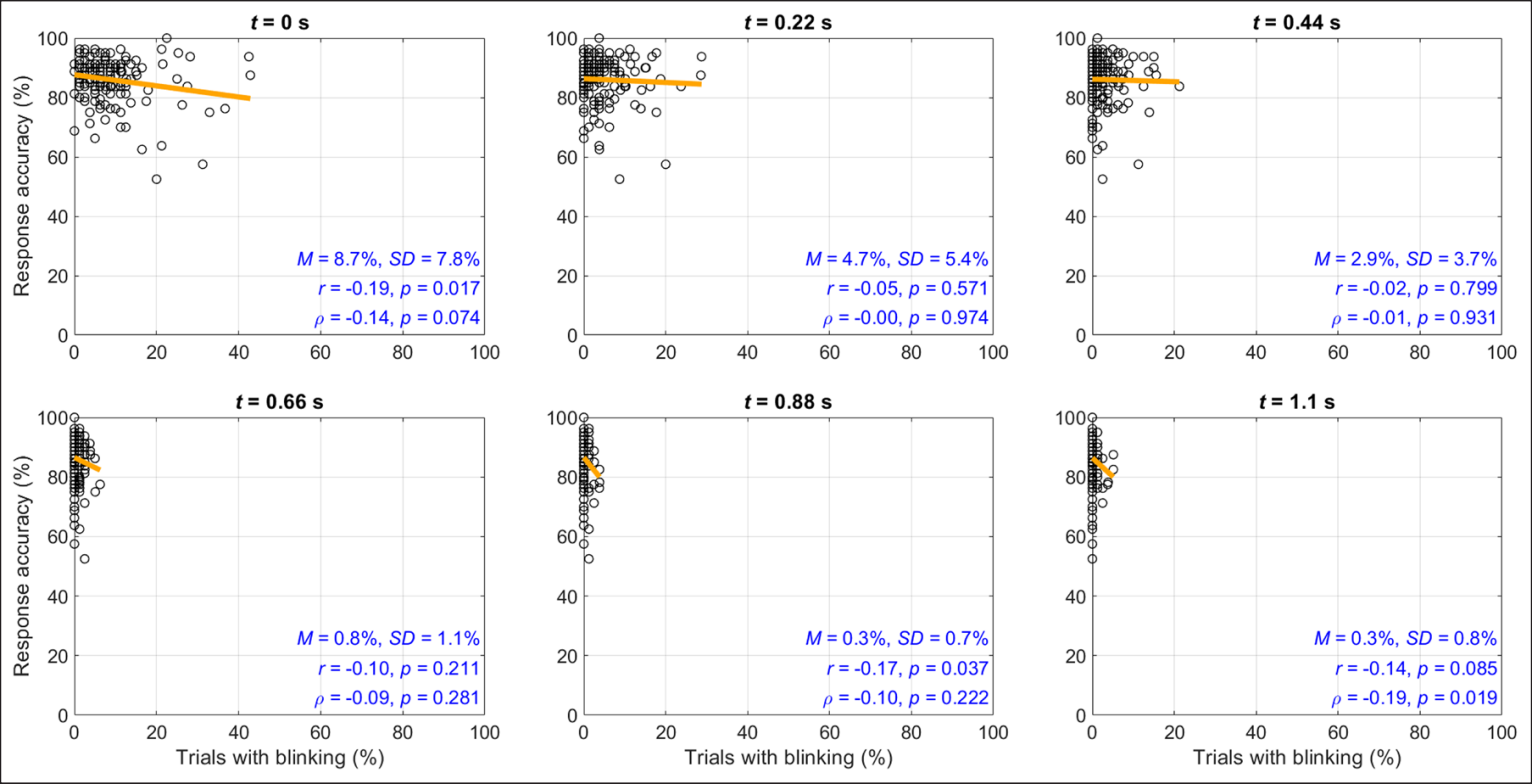
Follow-up experiment: Response accuracy (i.e., percentage of trials with a correct response) versus the percentage of trials with blinking at the level of participants (*N* = 159), per 220 ms of elapsed time into the trial. Also shown is a least-squares regression line, means and standard deviations of the percentage of trials with blinking, and the Pearson correlation coefficients (*r*) and Spearman rank-order correlation coefficients (ρ). The fixation marker onset occurs at *t* = 0 s. The onset of the Pi-figure occurs at *t* = 1.0 s.

However, the associations, as shown in Figure 9, were weaker as compared to the first experiment. It can also be seen from Figure 9 that there were only few trials in which participants blinked when the Pi-figure was shown (0.3% at 0.88 s and 0.3% at 0.3% at 1.1 s) in comparison to the first experiment (1.3% at 0.88 s and 0.5% at 1.1 s).

Finally, it was found that the overall response accuracy on the IT task (*M* = 86.04%, *SD* = 7.73%) correlated significantly (*r* = 0.18, *p* = 0.027) with the number of items that participants got correct on the Raven matrices (*M* = 7.30, *SD* = 1.93). This finding demonstrates some validity of the IT task as a predictor of performance on the Raven matrices task.

## Discussion

### Experience Effects and Overall Performance

The results of the first experiment showed that IT performance improved with trial number. In the follow-up experiment with improved task instructions and the inclusion practice trials, learning curves were still present. The shapes of the experience curves suggest that participants, in the aggregate, required about ten trials to get familiar with the task, after which they increased their attention to the task and reduced their response latency. The observed experience curves match previous research showing that the IT of children improves across sessions and testing days (Nettelbeck & Vita, 1992). Similarly, Bors et al. (1999) and Blotenberg and Schmidt-Atzert (2019) found that participants performed better when completing the IT session for a second or third time as compared to the first time.

Participants were aided with knowledge-of-results feedback, which can be expected to have contributed to improved performance as compared to not receiving such feedback (Salmoni, Schmidt, & Walter, 1984). Also, our study was conducted with MSc students at an engineering university, who are expected to have above-average IQs, presumably in the 115–130 range (based on Wai, Lubinski, & Benbow, 2009).

Despite the task feedback and presumably high intelligence of participants, performance in the first experiment was low, with a response accuracy of 86.2% for the highest exposure time. In addition, there were a considerable number of non-responses, especially in the first few trials. The low accuracy as well as non-responses can be explained by the fact that we provided participants with only basic instructions, no practice trials, and no performance feedback if the participants did not respond. In the follow-up experiment which included enhanced instructions, practice trials, and no response-time limit, a near-perfect response accuracy of 98.6% was obtained for the highest exposure time.

Our results point to the importance of making sure that participants understand the task. Previous IT studies have been conducted with different population groups, including children (e.g., Anderson, 1986; Nettelbeck & Young, 1990) and old persons (Johnson & Deary, 2011), which makes us wonder whether participants in all cases have understood the task. It seems plausible that the link between IT and IQ can, in part, be explained by the fact that persons with higher IQ are more likely to understand what they have to do while performing the IT task.

### Visual Illusions

The first experiment showed that IT performance is better among participants who reported a brightness illusion than among those who reported no illusion. These findings confirm previous research (e.g., Mackenzie & Bingham, 1985) regarding the benefit of perceiving illusions, with the difference that our study showed that the brightness illusion yielded a statistically significant benefit. In contrast, previous research was mostly concerned with the apparent movement illusion (see Introduction).

About 83% of participants who reported a visual illusion indicated using this illusion as a cue to perform the task. It is possible that participants intelligently deployed this cue for selecting the response key that was on the opposite side of the illusion. Egan (1994) explained: “Once the subject has become aware of this motion, s/he need only register the aftereffect, then press the response key on the side opposite to the region of motion.” (p. 307). The self-reports in the follow-up experiment indicated that participants did use such intelligent strategies, although the content of the responses varied considerably.

As pointed out above, visual illusions may cause one to employ a strategy that increases performance. However, our results suggest two additional explanations for the perception of illusions. First, the self-reports of the follow-up experiment indicate that what to consider an illusion is to some extent a matter of semantics. Some participants recognized the change from Pi-figure to the mask as a stretching/movement illusion, whereas other participants appeared to describe the same stretching/movements and did not regard it as an illusion, but merely as a change from one image to the other. Our observations appear to be in line with Simpson and Deary (1997) who found no causal effect of ‘macrolevel’ strategy use on IT and concluded that strategies are a verbalization of ‘microlevel’ cognitive processes.

A second explanation for the perception of illusions is that they are a by-product of understanding the task and knowledge of where to look. Conversely, if one does not understand the task or if one fails to distinguish the legs of the Pi-figure, then no illusion is likely to be perceived. An explanation for the superior performance of strategy users as an epiphenomenon has been considered before. Egan and Deary (1992), for example, argued that perceived illusions are “simply something seen when a discrimination is still possible for a subject at a short absolute IT duration” (p. 164). The standard deviations of the response accuracy, non-response percentage, and the mean response times were considerably smaller for the brightness illusion group as compared to the other two groups, which can be explained by the fact that a number of participants performed very poorly, sometimes around chance level (Figure S3a) or did not respond at all (Figure S5). These poor performers may have misunderstood the task or may have failed to see the legs of the Pi-figure. Spontaneous remarks by the participants reinforce the idea that the IT task was regarded as confusing. For example, a number of participants indicated that they thought they had to detect the difference in the lengths of the legs of the mask (while apparently not having seen the Pi-figure at all). In the follow-up experiment, we found no significant differences in task performance between four categories of strategy use versus no reported strategy use, and no incidences of extremely poor performance (Figure S3b). This finding reinforces the epiphenomenal explanation. In summary, the reporting of the brightness illusion may be a by-product of understanding the task or concentration at the task. It may even be hypothesized that the apparent motion illusion is a completely normal phenomenon that can be experienced by everyone, similar to the illusion of motion that occurs when playing the pictures of a movie at a minimal frame rate (Holcombe, 2009).

Alexander and Mackenzie (1992) reported four possible illusions: apparent motion, flash-brightness, ends-stand-out, and after-image, whereas Egan (1994) reported movement, flickering, and brightness. In our follow-up experiment, participants revealed interesting refinements to these illusions, with some referring, for example, to the fact that the short leg of the Pi-figure moved slower than the longer leg. Multiple-choice questions and free-response items, as used in the present study, provide only limited information about strategy use. For future research, we recommend performing interviews to examine how participants perceived and used the illusions. This recommendation is in line with Egan and Deary (1993), who advised “continuous monitoring of self-reports to describe the ‘on-line’ natural history of strategy development” (p. 135).

### Attention

Using eye-tracking equipment, we found that the IT task is highly dynamic: participants avoided blinking at the critical moment of the presentation of the Pi-figure. The overall increase in blinking with trial number, as shown in Figure 5, may have been caused by fatigue or eyestrain. In the first experiment, the correct/incorrect feedback (see Figure S2a) was bright and resulted in reflexive pupil constriction (see Figure S6), and may have contributed to a reflexive blinking response. However, in the follow-up experiment, with tight luminance control, many participants also blinked after the presentation of the Pi-figure, suggesting that this blinking is due to post-trial relaxation rather than due to a light reflex. In summary, participants in the first experiment and the follow-up experiment made sure that they were hardly blinking during the presentation of the Pi-figure, pointing to a crucial role of visual attention management while performing the IT task.

We found that whether one blinks at a particular moment of the trial was related to response accuracy. The corresponding correlations were stronger in the first experiment (ρ between –0.25 and –0.40) than in the follow-up experiment (ρ between –0.10 and –0.20). This difference can be explained by the larger individual differences in blinking and response accuracy in the first experiment, where some participants performed very poorly and blinked in a substantial number of trials when the Pi-figure was shown. Of note, the correlations are almost as strong as the correlation between IQ and IT, which Grudnik and Kranzler (2001) using meta-analysis estimated at –0.30 (uncorrected for range restriction and measurement error). Our IT-blinking correlations confirm early small-subject research of Nettelbeck et al. (1986), who found that a low-ability participant group (low-IQ participants, who obtained long IT scores) exhibited more blinking than a control group.

How should the correlation between blinking and IT be interpreted? On the one hand, it may be regarded as self-evident that blinking correlates with IT because if no light falls on the retina, better than chance performance is physically impossible. However, the blocking of light cannot be the only explanation of the observed IT-blinking correlations because only in a small number of trials (<1%) did the participants blink during stimulus presentation. Hence, blinks are not just a direct cause of poor IT performance, but also indicative of attention during the experiment in general. This is consistent with the above-mentioned epiphenomenal explanation of perceiving visual illusions: if not understanding the task or not knowing when/where the look, then blinking may be expected at inappropriate moments and performance may be expected to be poor.

Our work showed that IT is associated with motor activity of the eyelids, where motor activity refers to blinking after the presentation of the Pi-figure and blink inhibition when the Pi-figure is visible. The involvement of motor activity would be in contradiction to, amongst others, Jensen (2006), who stated that IT is captured “independently of the whole efferent aspect of RT” (p. 84). Not only blinking but also inhibition of blinking involves certain mental demands. An fMRI study by Chung Yoon, Song, and Park (2006) showed that voluntary and inhibited eye blinks involve the precentral gyrus, a region of the brain concerned with the coordination of movement. Berman Horovitz, Morel, and Hallett (2012) found, also using fMRI, that suppression of blinks is associated with a wide network of brain activations associated with the build-up of bodily urge.

### Conclusions and Recommendations

Our research contributes to the view that there is a multitude of factors associated with such a simple task as IT, including focused attention, the perception of illusions, understanding of the task, and task experience. These findings reject the hypothesis that IT is a univariate construct, and suggest that previously documented IT-IQ correlations are because of multiple overlapping processes (Kovacs & Conway, 2016; Spearman, 1923) rather than pure mental speed (see also Stankov, 2004).

A limitation of our study is that each participant completed only 80 IT trials and that long-term learning was not assessed. Another limitation is that our sample consisted of university students only. Although the use of university students appears to be common in IT research (Deary, Caryl, Egan, & Wight, 1989; Grudnik & Kranzler, 2001), a more heterogeneous sample can be expected to cause disattenuated correlations between IT and attention. Finally, it would be interesting to examine whether our findings regarding attention generalize to other types of elementary cognitive tasks. Johns et al. (2009) previously reported associations between blinking and visual reaction times. We expect that visual attention can explain a portion of the variance in task performance in psychometric tests.

In our follow-up experiment, we observed a modest correlation of 0.18 between IT and performance measured using a short version of Raven’s advanced progressive matrices. This correlation may become stronger if using a more heterogeneous pool of participants. Also, we recommend that future experiments include more participants and a full IQ test. It would be worthwhile to examine how task experience and blinking are associated with intelligence.

Finally, it would be useful to examine what display characteristics contribute to performance and criterion validity. Early studies used bright LED displays (Egan, 1994), whereas we used a grey background on a computer monitor. The use of computer screens has been criticized (Simpson & Deary, 1997), but display technologies have developed significantly over the last decades, now offering high refresh rates. It is possible that low contrast displays emphasize the factors the psychometrician is interested in, such as sensory speed, perceptual coding, or attentional processes (Levy, 1992). On the other hand, perhaps low contrast displays dilute the measurement of the speed of information intake as determined by, for example, nerve conduction velocity (Miller, 1994).

## Data Accessibility Statement

Raw data and materials are available at https://doi.org/10.4121/12961832.

## Additional File

The additional file for this article can be found as follows:

10.5334/joc.123.s1 Supplementary Materials. Additional tables and figures as cited in the text.
