# Supplementary material for: How Do People Perform an Inspection Time Task? An Examination of Visual Illusions, Task Experience, and Blinking
Source: J Cogn. 2020 Sep 30;3(1):34. doi: 10.5334/joc.123 (PMC7528665; doi:10.5334/joc.123)
Supplement: Supplementary Materials. — Additional tables and figures as cited in the text. [file joc-3-1-123-s1.pdf]

Supplementary Materials

Table S1

First experiment: Means (M) and standard deviations (SD) of performance measures

	**Response accuracy (%)**		**Response provided (%)**		**Mean response time (ms)**		**Median response time (ms)**	
**Exposure time (ms)**	*M*	*SD*	*M*	*SD*	*M*	*SD*	*M*	*SD*
14	52.79	19.70	91.50	16.85	1305	525	1179	538
21	55.60	19.60	92.65	14.68	1267	493	1151	527
35	69.35	21.46	94.08	13.64	1189	575	1079	618
42	74.68	23.52	94.49	13.76	1133	596	1020	615
56	81.96	20.66	94.76	13.91	1083	558	974	576
83	84.80	23.89	94.29	15.62	1062	612	979	650
104	85.87	22.38	95.44	13.15	1044	591	963	621
153	86.25	22.79	95.37	12.35	1049	563	958	580

Note. The results for ‘response accuracy’ and ‘mean response time’ were based on 146 or 147 participants. The results for ‘response provided’ were based on 147 participants.

Table S2

Follow-up experiment: Means (M) and standard deviations (SD) of performance measures (N = 159)

	**Response accuracy (%)**		**Median response time (ms)**	
**Exposure time (ms)**	*M*	*SD*	*M*	*SD*
14	57.58	19.03	785	343
21	69.18	19.26	711	256
35	86.10	15.13	610	203
42	90.12	11.96	605	196
56	93.13	11.39	564	169
83	96.66	7.61	547	162
104	97.04	7.59	548	150
153	98.55	6.74	562	182

Note. Because of anticipatory responses, response data were unavailable for 7 of 12720 trials.

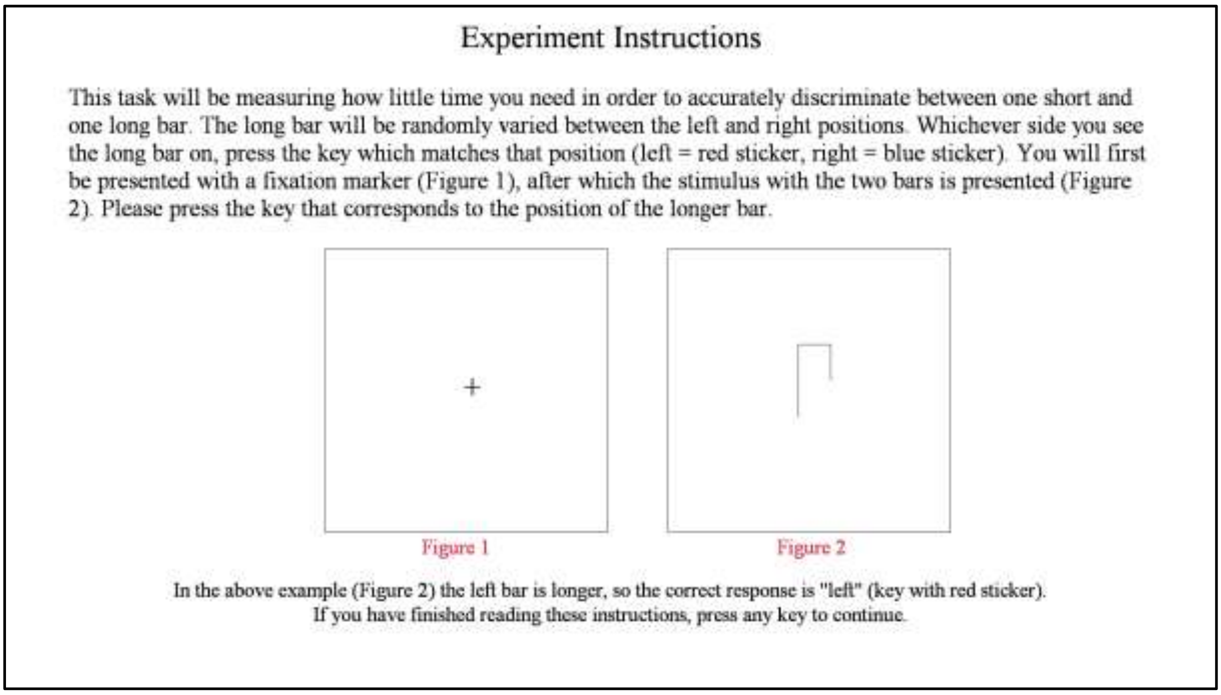

Figure S1. Instructions given prior to the experiment.

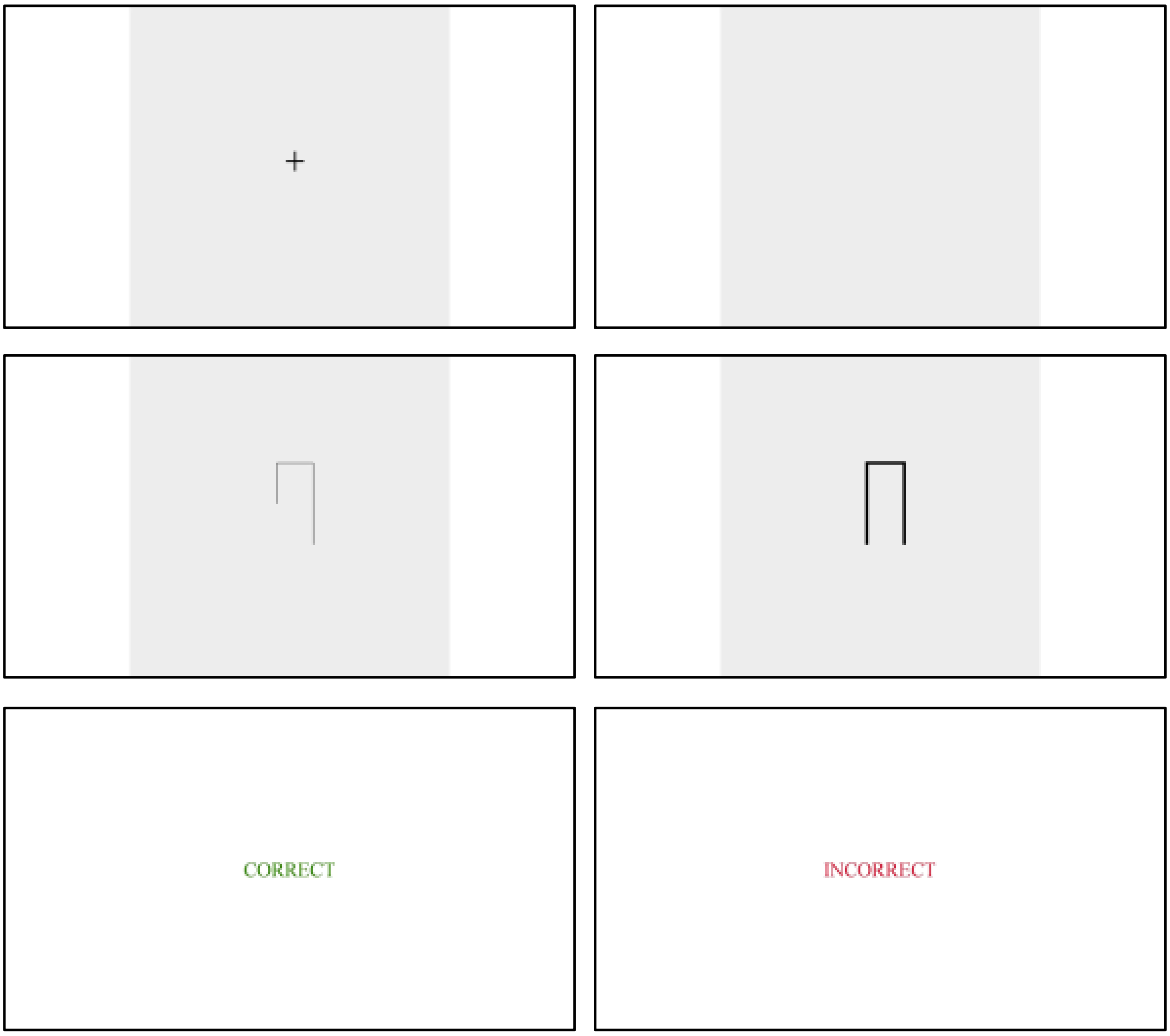

Figure S2a. First experiment: Components of an inspection time trial. Left top = Fixation marker, Right top = Blank screen, Left middle = Stimulus with one long and one short leg (i.e., the Pi-figure), Right middle = mask, Bottom left = Feedback after a correct response, Bottom right = Feedback after an incorrect response. The black figure outlines were not shown to the participants.

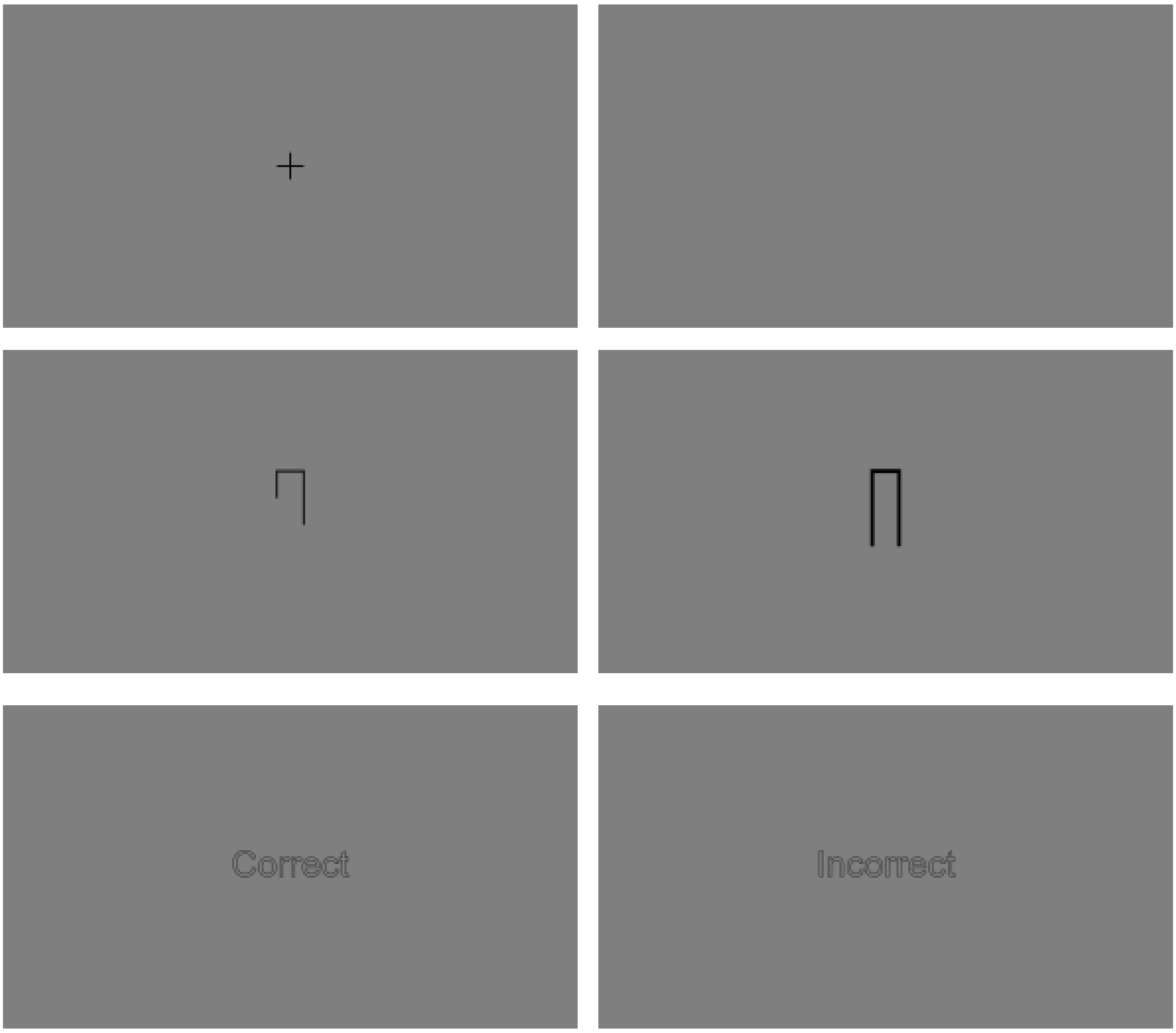

Figure S2b. Follow-up experiment: Components of an inspection time trial. Left top = Fixation marker, Right top = Blank screen, Left middle = Stimulus with one long and one short leg (i.e., the Pi-figure), Right middle = mask, Bottom left = Feedback after a correct response, Bottom right = Feedback after an incorrect response.

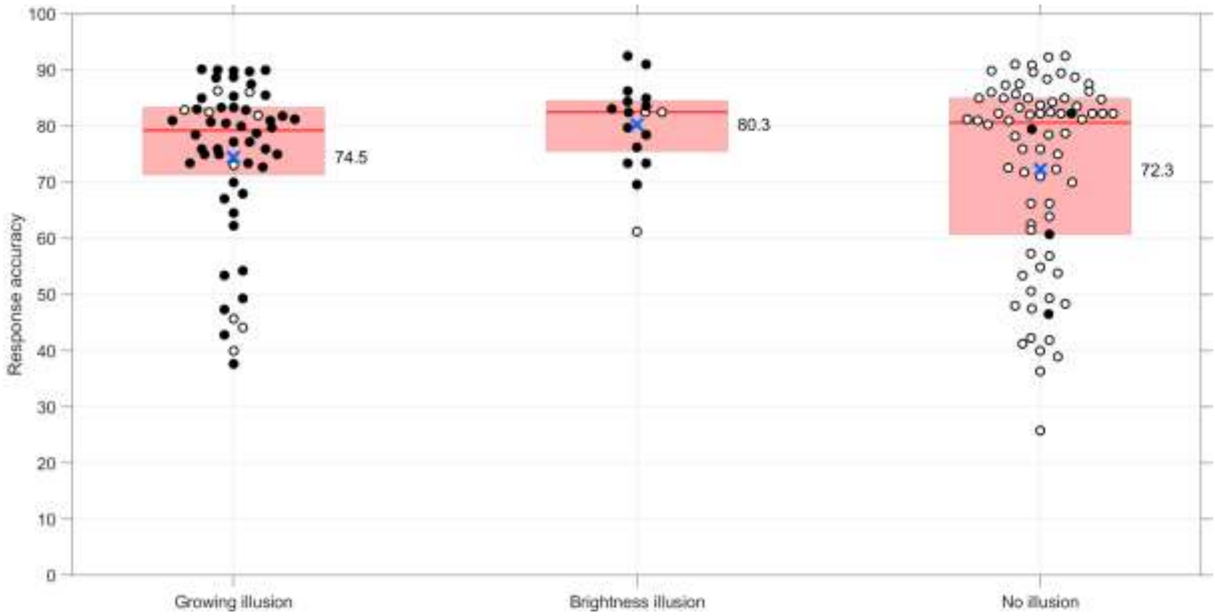

Figure S3a. First experiment: Response accuracy per participant as a function of reported illusion type. The boxplot shows the 25th percentile, median, and 75th percentile. The blue markers indicate the means. Black markers represent participants who reported ‘yes’ to the cue use question; white markers represent participants who reported ‘no’ to this question.

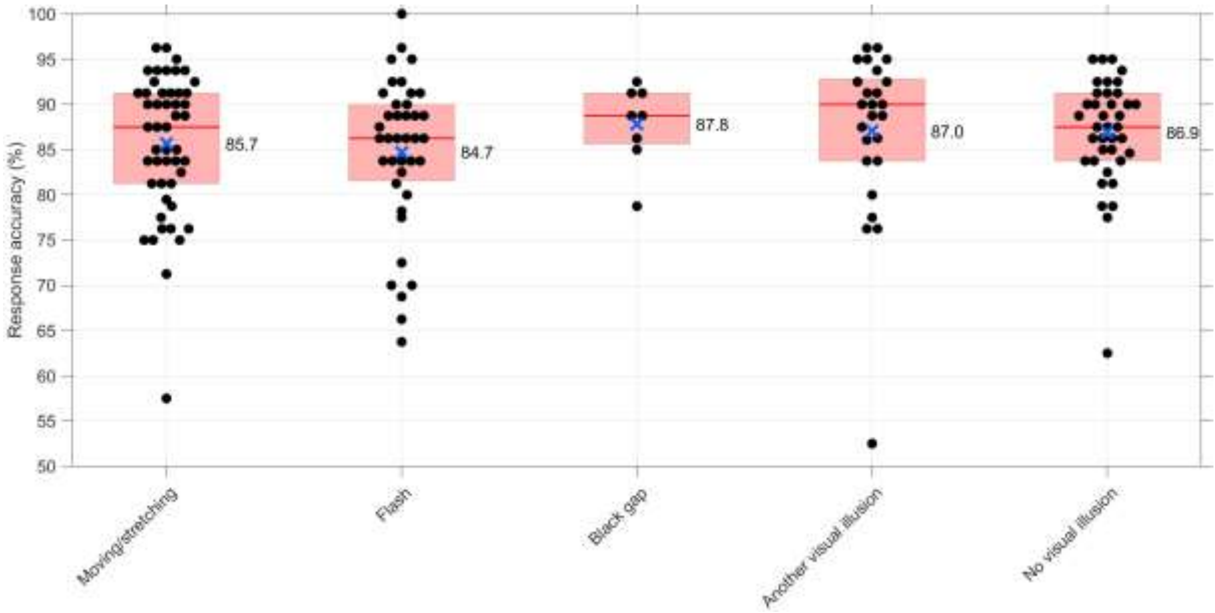

Figure S3b. Follow-up experiment: Response accuracy per participant as a function of reported illusion type. The boxplot shows the 25th percentile, median, and 75th percentile. The blue markers indicate the means.

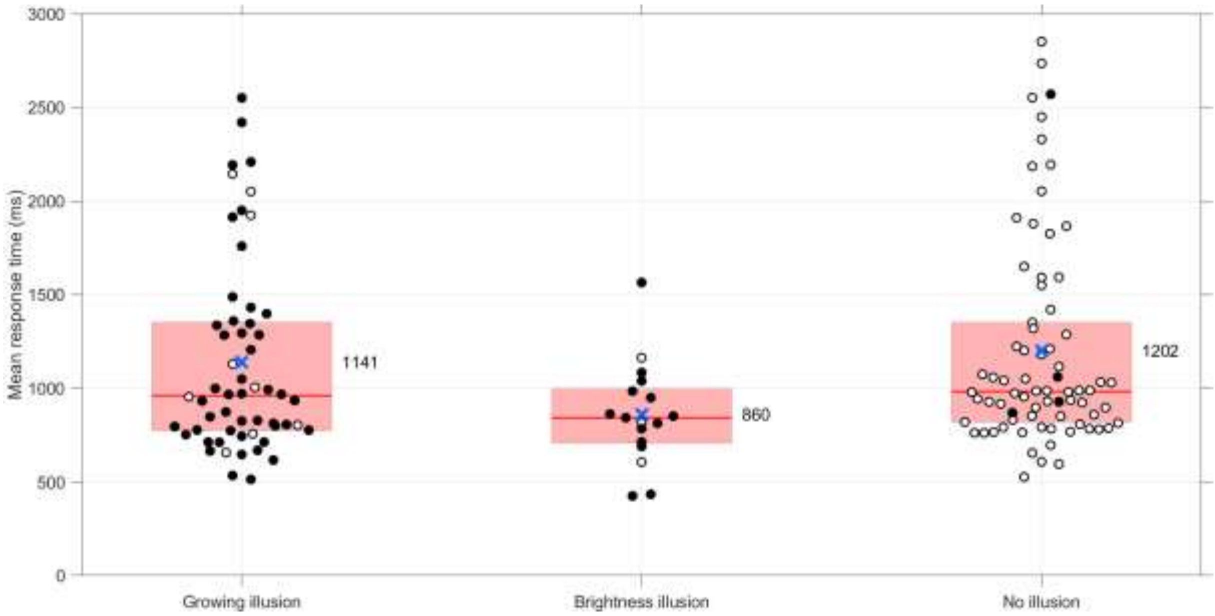

Figure S4a. First experiment: Mean response time per participant as a function of reported illusion. The boxplot shows the 25th percentile, median, and 75th percentile. The blue markers indicate the means. Black markers represent participants who reported 'yes' to the cue use question; white markers represent participants who reported 'no' to this question.

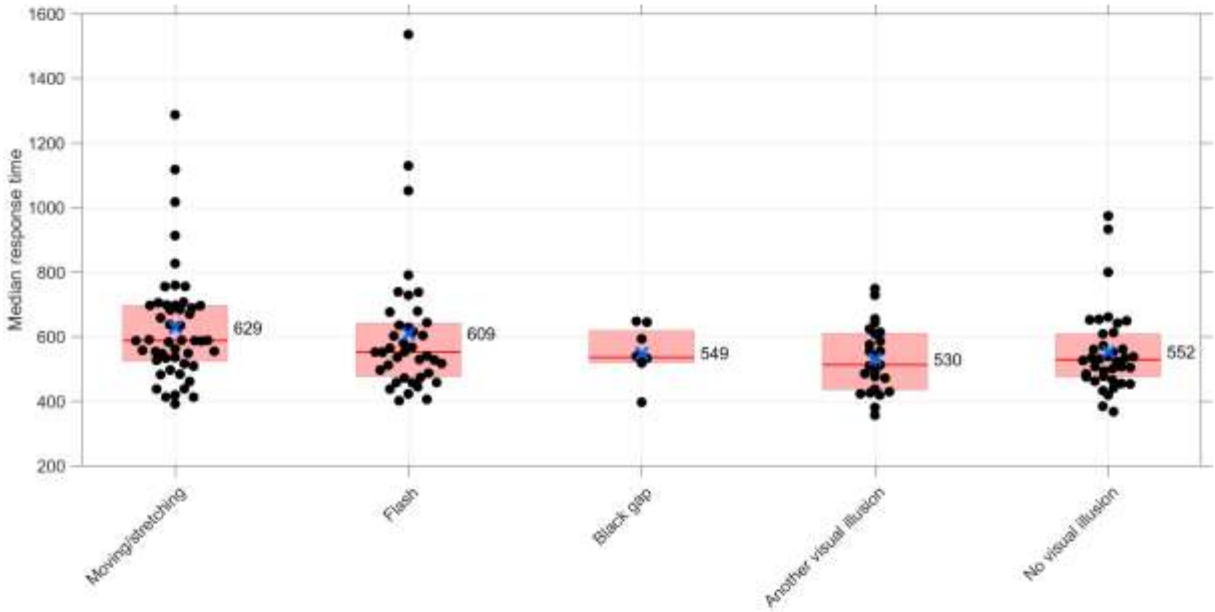

Figure S4b. Follow-up experiment: Median response time per participant as a function of reported illusion. The boxplot shows the 25th percentile, median, and 75th percentile. The blue markers indicate the means.

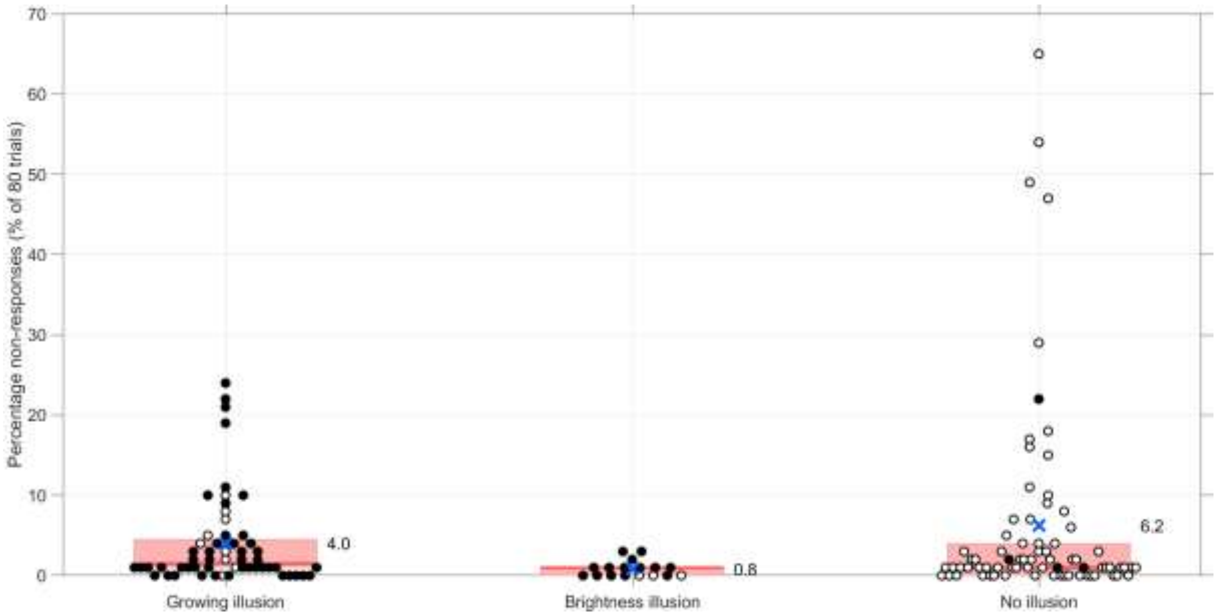

Figure S5. First experiment: Percentage of 80 trials in which the participant did not provide a response within the allocated time as a function of reported illusion type. The boxplot shows the 25th percentile, median, and 75th percentile. The blue markers indicate the mean. Black markers represent participants who reported ‘yes’ to the cue use question; white markers represent participants who reported ‘no’ to this question.

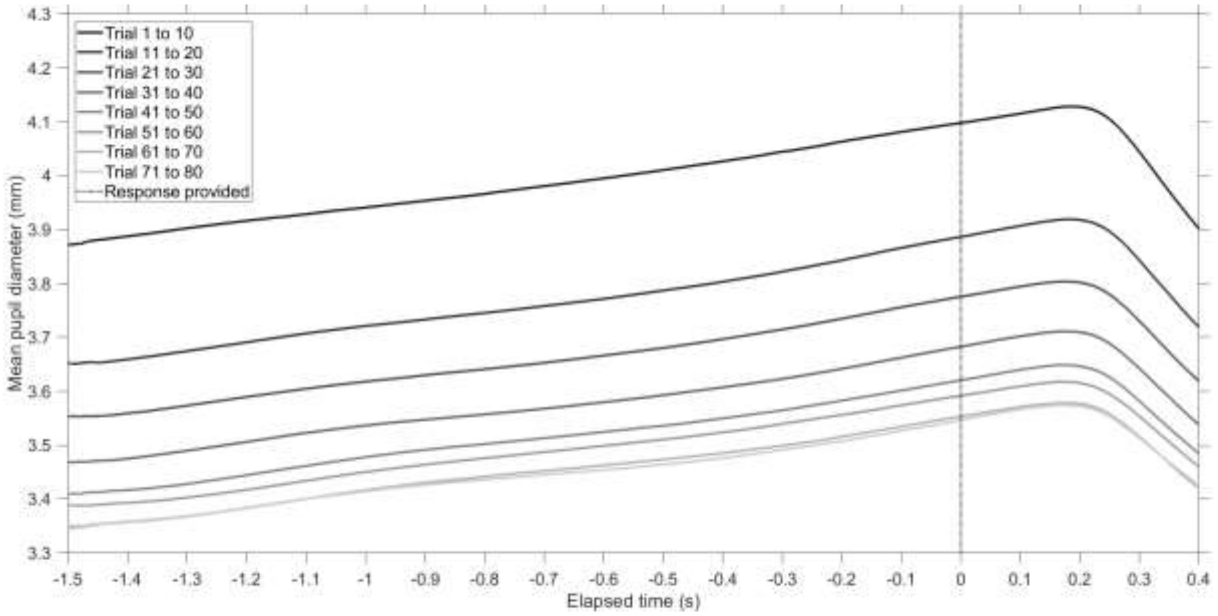

Figure S6. First experiment: Mean pupil diameter per group of 10 trials (11044 trials in total). The results are time-locked to the participants’ response (occurring at t = 0 s). Participants were provided with a “CORRECT” or “INCORRECT” feedback message after responding. Pupil diameter data were linearly interpolated during blinks. Data were included up to 0.4 s after the participant provided a response.

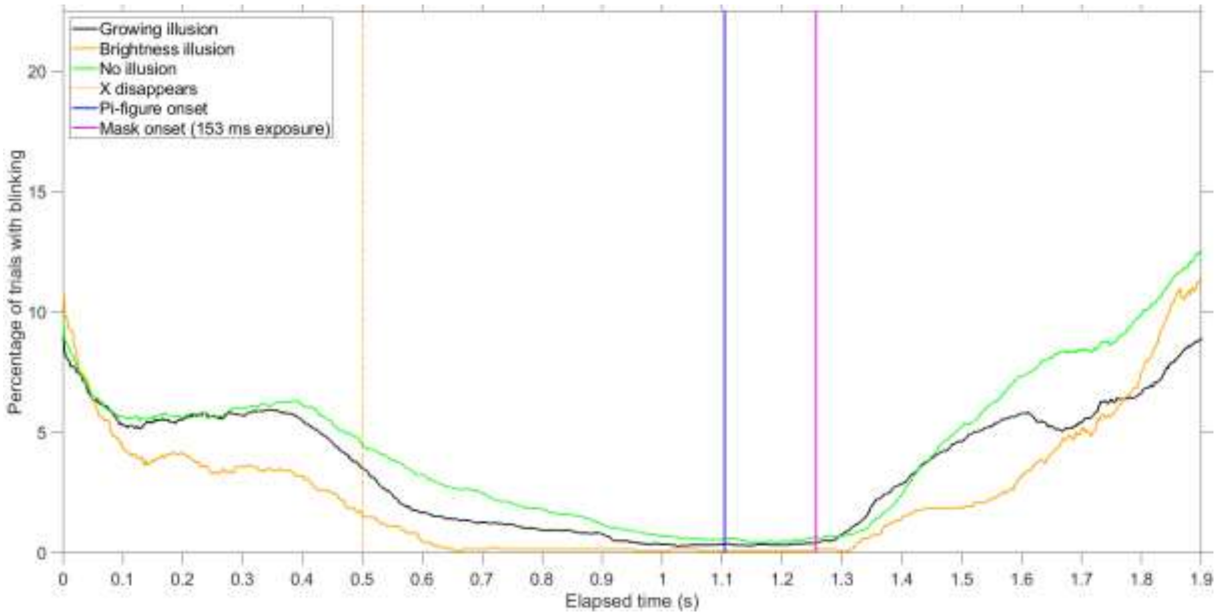

Figure S7. First experiment: Percentage of trials where the participant was blinking for the growing illusion (n = 4257), brightness illusion (n = 1345), and no illusion (n = 5442). Vertical lines are shown for the moment the fixation marker (X) disappeared, the moment the Pi-figure was presented, and the moment the mask was presented for the maximum exposure time of 153 ms. Data were included up to 0.4 s after the participant provided a response.
